# Do patients give a damn? An exploratory study of patient perspectives on clinician swearing during physical rehabilitation

**DOI:** 10.3389/fresc.2026.1719743

**Published:** 2026-10-01

**Authors:** Andrew Mercer, Adam Meakins, Roxanne St. Martin, Garrett Trummer, Libbi Geoghagan, Kara Muscha, Nicholas B. Washmuth

**Affiliations:** 1 Apex Performance and Wellness, Windsor, ON, Canada; 2 West Hertfordshire Teaching Hospital Trust, Hertfordshire, United Kingdom; 3 P4 Physical Therapy, Gardendale, AL, United States; 4 Outlaw Physio, Liberty Park, AL, United States; 5 Department of Physical Therapy, Samford University, Birmingham, AL, United States; 6 Department of Psychology, The University of Alabama in Huntsville, Huntsville, AL, United States

**Keywords:** adherence, chiropractic, motivation, physical therapy, professionalism, self-confidence, swearing

## Abstract

**Background:**

Swearing is a common linguistic tool associated with positive benefits. In healthcare, however, the impact of clinician swearing on patients remains unclear. This exploratory study investigated patients' lived experiences with clinicians who swore and examined patient perspectives on when clinician swearing is perceived as appropriate or inappropriate.

**Method:**

A cross-sectional, mixed methods, retrospective study was conducted with physical rehabilitation patients (*N* = 57). Patients completed an *ad hoc* survey assessing experiences, perceptions, and attitudes toward clinician swearing. Logistic regressions tested whether clinician swearing frequency predicted the following patient reported outcomes: motivation, self-confidence, and treatment adherence. Linear regressions evaluated whether clinician swearing frequency predicted: professionalism, patient-centeredness, offensiveness, and ability to enhance the rehabilitation experience. Qualitative responses were analyzed using cognitive network analyses.

**Results:**

Thirty-one patients (54.39%) reported that their clinician swore during treatment. Among these, most indicated swearing increased motivation (61.13%), improved self-confidence (58.06%), and supported adherence (51.61%). Patients generally agreed that swearing was patient-centered (54.84%) and improved the rehabilitation experience (64.52%), while no patients stated the swearing was unprofessional or offensive. When swearing frequency was dichotomized into high vs. low, patients in the high-frequency group reported greater motivation, self-confidence, and adherence compared to the low-frequency group, though confidence intervals included null effects. However, the small sample size limited statistical power; therefore, these nonsignificant findings should be interpreted cautiously. High-frequency swearing was associated with higher ratings of patient centeredness [*B* *=* −1.00, 95% CI (−1.95, −0.05), *p* = 0.04]. Qualitative findings indicated swearing was considered appropriate when used to build rapport, comfort, or motivate, and inappropriate with young or older patients, when directed at rather than with patients, or during serious discussions.

**Conclusion:**

Due to the study's limitations, the findings should be interpreted with caution. Most patients who experienced clinician swearing reported neutral to positive outcomes, suggesting swearing may enhance aspects of rehabilitation. Patients emphasized that appropriateness depends on context, relationship, and patient characteristics, suggesting that swearing may be a potentially effective communicative tool best applied with sensitivity.

## 1 Introduction

Swearing, defined as the use of taboo or potentially offensive language (1), is a nearly universal aspect of human communication (2). Though often regarded as inappropriate or unprofessional, swearing is both frequent and culturally embedded. For example, 58% of people report swearing “sometimes” or “often” (1), over 70% of adults say they frequently or occasionally hear others swear in public (3), and 57% of employees acknowledge swearing in the workplace (4). These findings suggest that swearing is increasingly normalized, even in formal or professional settings.

Beyond its controversial reputation, swearing is a linguistic tool that can serve a range of communicative functions and is associated with positive psychological, physiological, and social benefits (5). Swearing can foster informal environments that promote authenticity (6), build rapport (7), signal solidarity (8), enhance humor (1), and even increase perceptions of credibility (9). Additionally, swearing may boost self-confidence and improve task engagement (10).

In healthcare, the therapeutic bond between provider and patient is a well-established predictor of treatment adherence, satisfaction, and clinical outcomes (11–14). Given the social functions of swearing, especially its potential to express empathy and foster connection, strategic swearing by healthcare providers may offer a novel way to strengthen the therapeutic bond (15). However, the effects of clinician swearing on patient outcomes remain poorly understood. Evidence suggests both risks and benefits. Perrault et al. (16) found that patients rated doctors who swear lower in satisfaction, expertise, trustworthiness, and approachability. Yet, those same patients also perceived the swearing physicians as more human and relatable, and many said they would still choose to see that doctor again (16). In mental health settings, Giffin (17) surveyed 50 patients whose therapists had used swear words, and found overwhelmingly positive patient responses: 92% of patients reported that therapists should be able to swear around their clients, 80% reported that their therapist's use of swear words helped their therapeutic relationship, and over 88% of patients described an explicitly positive experience with therapist swearing. Notably, no patients reported that swearing harmed their therapeutic relationship (17).

In physical rehabilitation, the evidence remains ambiguous. Trummer, Stephens, and Washmuth (18) described two cases in which a physical therapist used strategic swearing to motivate patients and enhance their social connection. The outcomes were mixed, with one patient reporting improved therapeutic alliance and increased motivation, while the other reporting a slight decrease in perceived alliance. Importantly, neither patient found the swearing by the physical therapist to be offensive or unprofessional, and both patients viewed swearing as acceptable in clinical settings. These findings highlight the role of individual differences and contextual factors in shaping patient responses to clinician swearing.

Building on these insights, Washmuth, Meakins, Trummer, and Stephens (15) proposed that strategic, context-sensitive swearing may function as a patient-centered and professional communication tool. In appropriate contexts, swearing may reduce social distance, express empathy, and strengthen rapport, particularly when aligned with patient values and emotional needs. Nevertheless, empirical research on swearing in real-world clinical settings is sparse. Little is known about how swearing influences outcomes such as motivation, confidence, adherence, or clinical outcomes. To address these gaps, the present exploratory study investigates patients' lived experiences with clinicians who swore during physical rehabilitation. This exploratory study also examined patient perspectives on when clinician swearing is perceived as appropriate and when it is perceived as inappropriate. Specifically, this exploratory study addresses the following research questions:

RQ1. What is the prevalence of clinician swearing in physical rehabilitation settings?

RQ2. How does clinician swearing impact the patient?

RQ3. When do patients believe it is appropriate for a clinician to swear with a patient?

RQ4. When do patients believe it is not appropriate for a clinician to swear with a patient?

## 2 Materials and methods

### 2.1 Participants

A total of 64 patients were recruited using a convenience sampling method from three physical rehabilitation clinics: two physical therapy clinics located in Alabama, USA, and one chiropractic clinic in Ontario, Canada. The aim was to recruit as many patients as possible over a 10-month period, from August 2024 to May 2025. Clinicians at each site invited all patients aged 18 years or older to complete an anonymous survey on their discharge date (i.e., their final day of treatment). The 64 recruited patients were representative of the broader patient population across the three clinics; however, there was potential for selection bias, as patients may have chosen to participate because the topic applied to them. Only patients who had recently been discharged from care were invited to participate, based on the following rationale:

1. *Proximity to the care episode*: Recent discharge ensured that patients' recollections of their experiences were more accurate and detailed than those of patients surveyed weeks or months later.
2. *Reduced social desirability bias*: Patients were no longer receiving care at the time of the survey, which may have increased their willingness to response honestly without concern for potential effects on their treatment.
3. *Feasibility and engagement*: Discharge surveys are common practice in physical rehabilitation clinics, which may have improved patient participation.

Surveys were distributed via a secure Qualtrics link (Qualtrics LLC; Seattle, WA, USA). Data from seven patients were excluded due to missing responses on key survey items, resulting in a final sample of 57 patients (34 females, 23 males), with a mean age 40.39 years (*SD* = 16.00). All patients provided informed consent prior to participation. This study was approved by the Samford University Institutional Review Board (approval number EXMT-HP-24-F-1).

### 2.2 Design

A cross-sectional, mixed methods, retrospective survey design was utilized. An *ad hoc* instrument was created to address the four research questions (RQ1–4) by measuring patient experiences, perceptions, and attitudes related to clinician swearing during physical rehabilitation.

### 2.3 Materials

#### 2.3.1 Patient survey

A 14-item *ad hoc* survey was specifically developed for this study to assess patients' experiences with, and perceptions of, clinician swearing in physical rehabilitation settings (see full survey in the Open Science Framework repository: https://osf.io/4bu9h/). The survey, which included both scale and categorical items, aimed to evaluate the perceived impact of clinician swearing on patients (e.g., “In your opinion, what effect did your clinician's swearing have on your motivation?”) and to identify contextual factors that influence whether such language is viewed as appropriate or inappropriate (e.g., “In your opinion, when is it not appropriate for a clinician to swear with a patient?”). The survey items were informed by prior work on the interpersonal pragmatics of swearing in healthcare, including frameworks and findings described by Giffin (17); Trummer, Stephens, and Washmuth (18); and Washmuth, Meakins, Trummer, and Stephens (15).

### 2.4 Procedure

The study was introduced to patients under the title “When clinicians swear, do patients care?” At the time of discharge, clinicians invited eligible patients to participate by providing them with a quick-response (QR) code linked to the online survey. Patients completed the survey independently using their personal smart devices. The survey was distributed by clinicians who had previously acknowledged comfort with strategic swearing in clinical practice, which increased the probability that patients in this sample had been exposed to clinician swearing.

### 2.5 Statistics

Quantitative data were analyzed using IBM SPSS (version 28.0.1.1). Regression analyses were conducted to determine whether swearing frequency predicted patient outcomes. A series of logistic regression analyses were conducted to determine whether clinician swearing frequency (high vs. low) predicted each of the following patient reported outcomes: motivation, self-confidence, and treatment adherence. Due to the low number of patients reporting that their clinician used swear words (*N* = 31), clinician swearing frequency was dichotomized into two categories: high swearing frequency (more than once per session) and low swearing frequency (once per session or less). This categorization ensured at least 10 observations per category: high clinician swearing frequency (*N* = 19) and low clinician swearing frequency (*N* = 12). Motivation, self-confidence, and treatment adherence were coded as 1 = *increased* and 0 = *didn't affect*, as no patients indicated that these outcomes decreased in response to clinician swearing. Additionally, linear regression analyses were conducted to evaluate whether clinician swearing frequency (high vs. low) predicted perceptions of the swearing itself, specifically whether swearing was perceived as unprofessional, patient-centered, offensive, and as enhancing the rehabilitation experience. The criterion for statistical significance was set at an alpha level of *p* < 0.05.  Given the low number of patients in each group, the study was underpowered to detect anything but large effects. In underpowered studies, hypothesis testing using *p*-values can be unreliable; that is, *p*-values in such studies can show significance when there is none, and/or exaggerate the effect size of significant findings. Because of these concerns, and because the current study is exploratory, descriptive statistics and confidence intervals (CIs) are emphasized over *p*-values (19, 20) and can be used to inform *a priori* hypotheses in future research. A 95% CI represents a range of values in which a true population estimate (e.g., *B* in regressions) lies; if the same procedures are repeated across multiple studies, 95% of CIs would include the true estimate. A narrow range in a CI suggests precision, whereas a wide range suggests uncertainty in the true estimate.

Qualitative analyses were conducted using the Text Analytics Toolbox in MATLAB (version 2023a; MathWorks, Natick, MA). Pathfinder analysis (21) processed patients' open-ended responses into single-word terms. Important terms were then selected based on the frequency at which they occurred, with high frequency terms being more important than low frequency terms. After term selection, any like terms were combined (e.g., “doctor” and “clinician”). Cognitive networks were then created from the finalized terms, with these terms appearing as nodes connected to one another by links. Patients' open-ended responses were viewed to assist in the interpretation of the networks. Overall, the resulting networks depict important concepts (i.e., terms) in patients' open-ended responses, as well as the associations between concepts (i.e., the links).

### 2.6 Transparency and openness

The full *ad hoc* survey used in this study and the dataset presented can be found in the Open Science Framework repository: https://osf.io/4bu9h/.

## 3 Results

### 3.1 RQ1. What is the prevalence of clinician swearing in physical rehabilitation settings?

Descriptive and frequency data are presented in Table 1. Among the 57 patients surveyed, 31 (54.39%) reported that their clinician used swear words during their sessions. Of these 31 patients, three (9.69%) indicated that their clinician swore only once, four (12.90%) reported their clinician swore less than 1x/visit, five (16.13%) reported their clinician swore 1x/visit, and 19 (61.29%) reported their clinician swore more than 1x/visit.

**Table 1:** Descriptive and frequency data from *ad hoc* survey.

Survey items	*N* (%)
Did your clinician use swear words at all during your sessions? (*N* = 57)	
Yes	31 (54.39%)
No	26 (45.61%)
From patients who experienced clinician swearing (*N* = 31):	
How often did your clinician swear?	
1 time only	3 (9.68%)
Less than 1x/visit	4 (12.90%)
1x/visit	5 (16.13%)
More than 1x/visit	19 (61.29%)
What effect did your clinician's swearing have on your motivation?	
Increased motivation	19 (61.13%)
Didn't affect motivation	12 (38.71%)
Decreased motivation	0 (0.00%)
What effect did your clinician's swearing have on your self-confidence?	
Increased self-confidence	18 (58.06%)
Didn't affect self-confidence	13 (41.94%)
Decreased self-confidence	0 (0.00%)
What effect did your clinician's swearing have on your adherence to treatment?	
Increased adherence	16 (51.61%)
Didn't affect adherence	15 (48.39%)
Decreased adherence	0 (0.00%)
Your clinician's swearing was unprofessional	
Strongly agree	0 (0.00%)
Agree	0 (0.00%)
Neither agree nor disagree	6 (19.35%)
Disagree	10 (32.26%)
Strongly disagree	15 (48.39%)
Your clinician's swearing was patient-centered	
Strongly agree	11 (35.48%)
Agree	6 (19.35%)
Neither agree nor disagree	8 (25.81%)
Disagree	3 (9.68%)
Strongly disagree	3 (9.68%)
You were offended by your clinician's swearing	
Strongly agree	0 (0.00%)
Agree	0 (0.00%)
Neither agree nor disagree	1 (3.23%)
Disagree	5 (16.13%)
Strongly disagree	25 (80.65%)
Your clinician's swearing improved your experience with physical rehabilitation	
Strongly agree	13 (41.94%)
Agree	7 (22.58%)
Neither agree nor disagree	10 (32.26%)
Disagree	1 (3.23%)
Strongly disagree	0 (0.00%)

### 3.2 RQ2. How does clinician swearing impact the patient?

Among the 31 patients whose clinician swore during treatment, most reported positive or neutral outcomes. Specifically, the majority indicated that swearing increased their motivation (61.13%), improved their self-confidence (58.06%), and supported adherence to treatment (51.61%). Furthermore, most patients agreed or strongly agreed that the clinician swearing was patient-centered (54.84%) and improved their experience with physical rehabilitation (64.52%), and most patients disagreed or strongly disagreed that the clinician swearing was unprofessional (80.65%) and offensive (96.77%). See Table 1 for descriptive and frequency data.

Clinician swearing frequency of the 31 patients who experienced clinician swearing was dichotomized into two categories: high swearing frequency (more than once per session) and low swearing frequency (once per session or less). Logistic regressions were used to analyze categorical data, and linear regressions were used to analyze continuous data. Results suggested that patients felt more motivated, more self-confident, and had higher treatment adherence when clinician swearing frequency was high compared to low. Specifically, more patients in the high clinician swearing frequency group indicated that swearing increased their motivation (68.42%) compared to those in the low clinician swearing frequency group (50.00%), *B* = 0.77, OR = 2.17, 95% CI [0.45, 9.60], *p* = 0.31. Similarly, more patients in the high clinician swearing frequency group indicated that the swearing increased their self-confidence (63.16%) than those in the low clinician swearing frequency group (50.00%), *B* = 0.54, OR = 1.71, 95% CI [0.40, 7.43], *p* = 0.47. Lastly, more patients in the high clinician swearing frequency group indicated that swearing increased their adherence to treatment (63.16%) than those in the low clinician swearing frequency group (33.33%), *B* = 1.23, OR = 3.43, 95% CI [0.75, 15.67], *p* = 0.11. The CIs for each dependent measure span across a wide range of values, including null effects.

Regarding the linear regression analyses, the mean differences between the high and low clinician swearing frequency groups were small regarding ratings of unprofessionalism (*M_D_* = −0.21), *B* *=* −0.21, 95% CI [−0.80, 0.39], *p* = 0.49, and offensiveness (*M_D_* = 0.04), *B* *=* 0.04, 95% CI [−0.34, 0.42], *p* = 0.83. The difference between swearing frequency groups was slightly higher for ratings of how clinician swearing impacted the rehabilitation experience and patient-centeredness. Those in the high clinician swearing frequency group felt that swearing improved their rehabilitation experience relative to those in the low clinician swearing frequency group, (*M_D_* = -.59), *B* *=* −0.60, 95% CI [−1.29, 0.10], *p* = 0.09. The CIs for these ratings span a wide range of values, including null effects. Regarding patient-centeredness, patients in the high clinician swearing frequency group rated clinician swearing as being more patient-centered than those in the low clinician swearing frequency group (*M_D_* = −1.00), *B* *=* −1.00, 95% CI [−1.95, −0.05], *p* = 0.04. Although the CI for patient-centeredness does not include null effects, the range of values is wide.

### 3.3 RQ3. When do patients believe it is appropriate for a clinician to swear with a patient?

In responses to the question, “In your opinion, when it is appropriate for a clinician to swear with a patient?”, the 57 patients that completed the survey focused on the impact swearing would have on the patient (see Figure 1a). Patients thought that swearing is appropriate when it benefits the patient, such as if the swearing is (a) not harmful to the patient, (b) used to emphasize a point, and/or (c) used to build rapport, comfort, and motivation. Patients also thought that the appropriateness of clinician swearing depends on the clinician's relationship with the patient.

**Figure 1:**
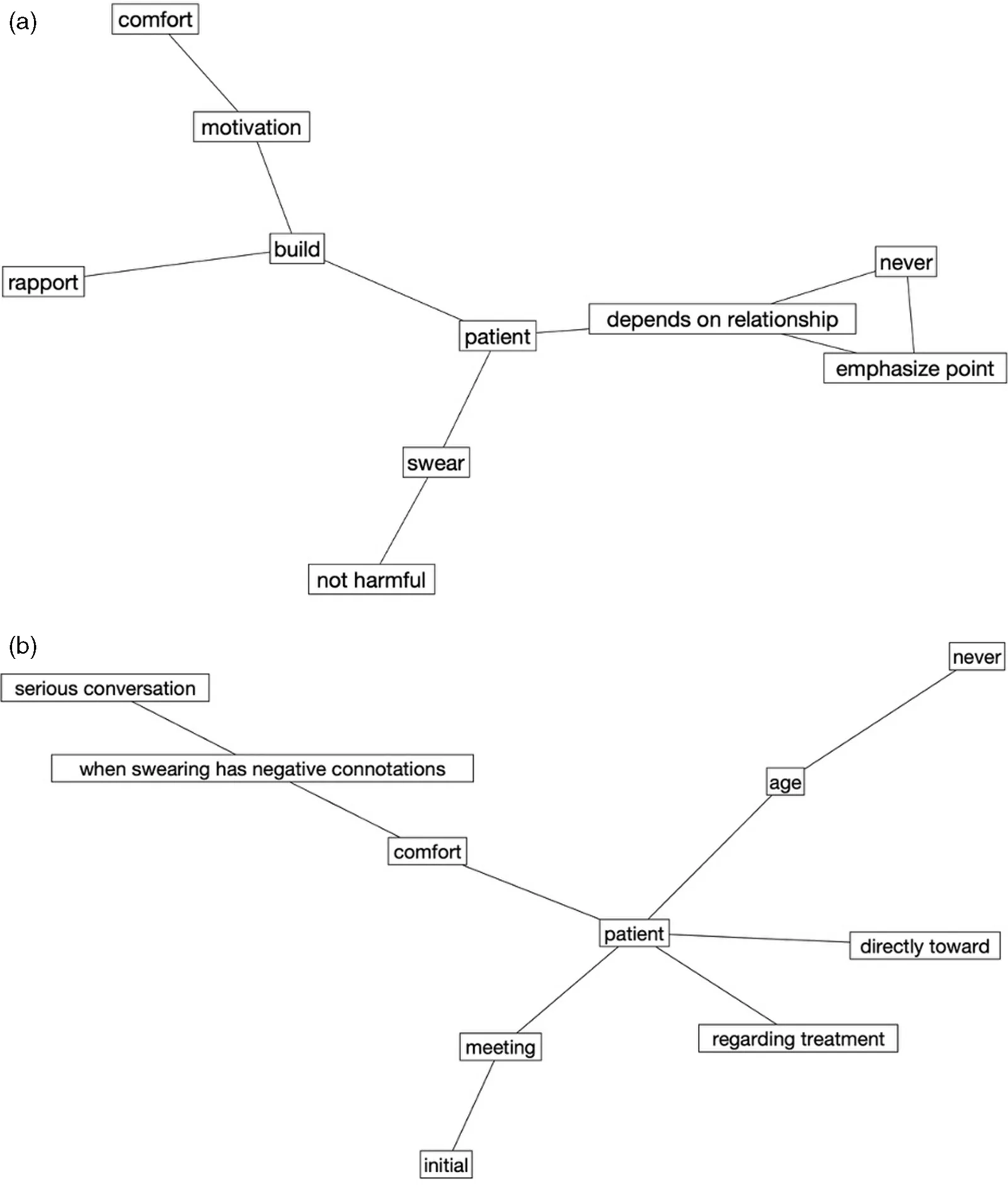
When patients believe clinician swearing **(a)** is appropriate and **(b)** is not appropriate.

### 3.4 RQ4. When do patients believe it is not appropriate for a clinician to swear with a patient?

Regarding the question, “In your opinion, when is it not appropriate for a clinician to swear with a patient?”, the 57 patients that completed the survey centered their responses on the individual characteristics of the patient. These characteristics included the patient's age (i.e., inappropriate with older patients) and the patient's comfortability with swearing (see Figure 1b). Further, patients thought that clinician swearing is not appropriate if it is *at* rather than *with* the patient, and/or if swearing is used in serious conversations (e.g., pertaining to the patient's treatment). Finally, patients believed that clinician swearing is not appropriate when it would be detrimental to the patient-clinician relationship.

Notably, in response to both questions (RQ3 and RQ4), some patients thought that clinician swearing was never appropriate (as seen in both networks in the nodes “never”).

## 4 Discussion

This study applied a cross-sectional, mixed methods, retrospective survey design to explore how clinician swearing is perceived by patients in physical rehabilitation settings. Specifically, this exploratory study examined patients' experiences, attitudes, and contextual judgments regarding when clinician swearing is appropriate or inappropriate. These findings contribute to a growing body of literature that seeks to understand the communicative, psychological, and relational effects of swearing in healthcare contexts.

This exploratory study aimed to examine patients' lived experiences with clinicians who swore and to gain insight from actual patients on when it is appropriate, and inappropriate, for a clinician to swear. As such, it was inherently exploratory in nature and purpose, guided by four research questions, which will serve as a framework for discussion.

### 4.1 RQ1. What is the prevalence of clinician swearing in physical rehabilitation settings?

Of the 57 patients surveyed, 31(54.39%) reported that their clinician used swear words during treatment. While this provides an initial estimate, the prevalence reported here may differ than the typical rates in rehabilitation settings, as the clinicians who distributed the survey acknowledged their comfort level with swearing during clinical care. General population prevalence data on clinician swearing remain scarce and difficult to obtain (22). Prior reports, such as Trummer, Stephens, and Washmuth (18), provide only case-level data and suggest that swearing in clinical contexts is relatively infrequent and situation-specific. Therefore, this study's prevalence data should be interpreted with caution and not generalized beyond the sample providers. Additionally, the geographic scope of this study was limited to two physical therapy clinics in the United States and one chiropractic clinic in Canada. Cultural attitudes toward swearing vary significantly across geographical regions (23), and it is likely that including European-based therapists and patients would yield different results.

### 4.2 RQ2. How does clinician swearing impact the patient?

Among the patients whose clinician swore during treatment, most reported positive or neutral outcomes (see Table 1). Specifically, the majority indicated that swearing increased their motivation, improved their self-confidence, and supported adherence to treatment. Furthermore, most patients agreed or strongly agreed that the clinician swearing was patient-centered and improved their experience with physical rehabilitation, and most patients disagreed or strongly disagreed that the clinician swearing was unprofessional and offensive. Notably, none of the patients stated that clinician swearing decreased their motivation, self-confidence, adherence to treatment, was unprofessional, or that they were offended by clinician swearing. It should be reemphasized here that the clinicians who distributed the survey acknowledged their comfort level with swearing during clinical care. Therefore, these clinicians may utilize swearing in more contextually appropriate ways than other clinicians. Nevertheless, these findings align with previous work in mental health (17), where patients overwhelmingly supported therapist swearing and reported no harm to the therapeutic relationship. Similarly, Trummer, Stephens, and Washmuth (18) found that patients exposed to clinician swearing perceived it as acceptable and not damaging to rapport. Taken together, these findings suggest that strategic, well-intentioned swearing, particularly when used to motivate, build solidarity, or express authenticity, can enhance the therapeutic experience with minimal risk of offense. However, context is key. Disciplinary actions have been taken against healthcare providers for swearing out of frustration or anger, highlighting that swearing used without patient-centered intent can produce negative outcomes (24, 25). Another important consideration is that different swear words may carry different levels of impact. For example, a therapist saying f**k vs. s**t vs. c**t is likely to be interpreted differently by patients, given variations in offensiveness across these terms (1).

#### 4.2.1 Motivation

There is ongoing debate regarding how best to define “motivation” in the context of patient behavior (26). Historically, motivation was conceptualized as a fixed, internal personality trait, something intrinsic to the individual and unaffected by external influences (27). In contrast, contemporary perspectives view motivation as dynamic and shaped by social and environmental factors. These include the rehabilitation setting, the clinician's attitude toward the patient, and the overall interpersonal climate of care (26, 28, 29). According to Maclean and Pound (26), clinicians who are perceived as kind, welcoming, and genuinely engaged in the patient's experience can positively influence patient motivation.

Swearing, when used appropriately, may contribute to such an environment. Swearing has been linked to tighter human bonds by fostering informal, authentic interactions in which individuals feel more comfortable being themselves (6). This informality may facilitate rapport (30) and communicate solidarity (8), reinforcing a sense of shared experience or “we're in this together.” In the present study, clinician swearing may have served as a social cue that created a personable and relaxed clinical atmosphere, which in turn enhanced patients' sense of motivation.

#### 4.2.2 Self-confidence

A common psychological response following injury is a decline in self-confidence, particularly regarding one's ability to return to full participation in activities (31). One influential factor in restoring confidence is verbal persuasion, or encouragement provided by a clinician, which is shaped not only by the message itself but also by the context and the perceived sincerity of the provider (32). When clinicians swear during encouragement [e.g., “H**l yes bro, that is good s**t! Keep up the f**king good work!”; (18)], they may be delivering a more intense and emotionally resonant form of verbal persuasion. Feedback that includes swearing may convey authenticity, enthusiasm, and solidarity, helping break through doubt and reinforce belief in one's ability.

The importance of self-confidence in rehabilitation cannot be overstated. Reduced confidence is associated with poorer functional outcomes after injury (33), suggesting that even subtle boosts in confidence can meaningfully affect recovery. Clinician swearing may also serve as implicit permission for patients to speak more freely themselves. In hierarchical healthcare interactions, where patients may otherwise feel constrained, the normalization of informal language can empower patients to express their authentic thoughts and emotions. The permission to be their authentic self may foster a stronger sense of trust and partnership with the clinician. Strategic swearing may therefore serve as a clinical tool, when used appropriately, to enhance patients' self-confidence and support their rehabilitation progress.

#### 4.2.3 Patient adherence

The success of physical rehabilitation is closely tied to patient adherence (34); however, non-adherence remains a common barrier to effective outcomes (35). Understanding how clinician behaviors, such as swearing, affect adherence may offer practical guidance on whether, when, and how swearing might be used to support patient engagement. Shahidi et al. (35) emphasize the central role of the therapeutic alliance, highlighting the importance of the clinician's interpersonal style, in patient adherence. Patients consistently value providers who are supportive, nonjudgmental, and relatable, traits that help foster a strong therapeutic connection. When used strategically, swearing may enhance social connectedness and allow clinicians to come across as more authentic and approachable. This, in turn, can strengthen the therapeutic alliance. In addition to relational factors, the tone and enjoyment of rehabilitation sessions also influence patient compliance (35). Swearing, if used with sensitivity to context and individual preferences, may inject humor and informality into the session, making the rehabilitation experience feel less sterile and more engaging. A lighter, more enjoyable atmosphere may boost mood, increase patient satisfaction, and ultimately encourage greater adherence to treatment plans.

#### 4.2.4 Clinician swearing frequency (e.g., dosage)

Dosage, defined in terms of intensity, frequency, or quantity, is a critical factor in many interventions. In strength training, for example, greater volume (e.g., quantity) is associated with increased muscular adaptations (36, 37), while in alcohol consumption, higher quantity and frequency are linked with greater health risks (38, 39). However, swearing, when conceptualized as a behavioral strategy or intervention to enhance outcomes such as motivation, self-confidence, treatment adherence, or perceptions of professionalism, appears to follow a different pattern.

In this exploratory study, higher swearing frequency was not reliably associated with stronger patient outcomes. Patients exposed to frequent clinician swearing (more than once per session) generally reported greater motivation, self-confidence, and adherence than those exposed to less frequent swearing, but the confidence intervals for these effects were wide and included null values. Importantly, frequent swearing did not increase perceptions of unprofessionalism or offensiveness. On the contrary, higher swearing frequency was linked with greater perceptions of patient-centeredness.

Several explanations may account for this pattern. It is possible that the presence of swearing, rather than the frequency, drives patient perceptions of motivation, confidence, and adherence. In other words, swearing may operate more as a threshold effect than as a dose-response effect. At the same time, patients' qualitative responses highlighted that appropriateness depends on context, timing, and the patient-clinician relationship. Swearing that feels tailored to the specific patient may strengthen rapport, while overuse or poorly timed swearing risks diminishing its perceived authenticity and relevance. The impact of swearing may also depend on the intensity of the words chosen (e.g., mild vs. strong swear words), which were not measured in the current exploratory study and could further complicate simple frequency effects.

### 4.3 RQ3. When do patients believe it is appropriate for a clinician to swear with a patient?

Patients emphasized that the appropriateness of clinician swearing was situational and largely dependent on its impact on the therapeutic relationship. Many patients noted that swearing was acceptable when it served to motivate, encourage, or affirm progress. For example, one patient wrote, “Motivational, affirmation, compliment”, while another described swearing as appropriate “in an attempt to motivate a patient.” These responses are consistent with prior case-level findings suggesting swearing can function as a form of intensified verbal persuasion, capable of boosting patient engagement and confidence (18).

Another theme was the role of rapport and relational context. Patients frequently described swearing as appropriate once a clinician had established trust: “depends on the relationship the [clinician] has w[ith] the patient” and “when he/she has built rapport with the patient.” This aligns with Giffin's (17) study in mental health settings, where patients highlighted the relational foundation as critical to interpreting swearing as supportive rather than inappropriate. Such findings also resonate with broader therapeutic alliance research, which emphasizes the importance of trust and connection in shaping patient perceptions of clinician behaviors (11, 12).

Some patients endorsed swearing when it was used for humor, empathy, or solidarity: “When empathizing with patients, joking, showing support.” These contexts are consistent with sociolinguistic findings that swearing can function to reduce social distance, express authenticity, and signal shared identity (6, 8). Taken together, these responses suggest that swearing is judged as appropriate when it is patient-centered, relationally grounded, and purpose-driven rather than habitual or careless.

### 4.4 RQ4. When do patients believe it is not appropriate for a clinician to swear with a patient?

Patients outlined boundaries for when clinician swearing was viewed as inappropriate. Patient characteristics were particularly salient, with many highlighting age: “kids under 10” and “underage patient”. Similarly, some patients expressed that swearing should be avoided with older adults, reflecting concerns about generational differences in tolerance of taboo language (1).

Another recurring theme was patient comfort and consent. One patient noted, “If the client is NOT comfortable with it,” while another said, “If a patient has explicitly stated so.” These findings reinforce the importance of aligning clinician language with individual patient preferences and values, consistent with principles of patient-centered care (15).

Patients also cautioned against swearing that was directed at the patient or used in serious clinical conversations. For example, respondents wrote: “In frustration, derogatory” and “when they are no longer swearing with and are swearing at [the patient].” This distinction mirrors previous reports of disciplinary action against healthcare providers who swore out of anger or in ways that undermined professionalism (24).

Finally, a subset of patients rejected clinician swearing outright, responding simply with “Never” or “Always [inappropriate].” This absolute rejection underscores that while many patients view swearing as conditionally acceptable, it remains unacceptable and inappropriate for others, highlighting the need for clinicians to exercise sensitivity and discretion.

### 4.5 Limitations and future research

While this exploratory study contributes to the growing understanding of how clinician swearing may impact patients and offer insights for when it may be appropriate or inappropriate for a clinician to swear with a patient, several limitations should be considered when interpreting the findings of this study. First, the use of a convenience sample introduces the potential for selection bias. The clinicians who distributed the survey were recruited specifically because they had previously acknowledged swearing in clinical practice, which likely inflated the observed prevalence of clinician swearing and may limit the generalizability of findings to other rehabilitation providers or settings. Second, the study relied on retrospective, self-reported data, which are susceptible to recall bias and social desirability effects. While efforts were made to enhance the accuracy and decrease bias (e.g., distributing survey immediately post-discharge), the possibility remains that patients underreported or overreported their reactions to clinician swearing. Third, the sample size, especially after dividing patients into high vs. low clinician swearing frequency groups, limited statistical power to detect subtle effects. As a result, the analyses were markedly underpowered, and findings should be interpreted with caution. Fourth, the lack of diversity in clinician participants and clinic types (limited to physical therapy and chiropractic settings) prevent generalizing the findings to other healthcare disciplines. Fifth, this study did not assess clinician intent, tone, or clinician-patient rapport prior to swearing, which would likely moderate how swearing is perceived. Without this contextual information, it is difficult to determine whether outcomes were driven by the swearing itself or by other factors present. Sixth, the qualitative analyses, while thematically informative, were based on only two open ended survey questions. More in-depth methods such as interviews or focus groups may yield richer insights into the judgments patients make regarding the appropriateness, or inappropriateness, of swearing.

Future studies should extend this exploratory work through preregistered, confirmatory studies using larger, more representative samples across diverse clinical settings and healthcare fields (e.g., nursing, oncology, pediatrics). Longitudinal and experimental designs should be employed to help determine causal relationships between clinician swearing and outcomes. Additionally, future studies should explore moderating variables, including patient demographics (e.g., age, gender, cultural background), personality traits, and communication preferences, as well as clinician-specific factors, such as tone, intent, specific swear words used (e.g., f**k vs. s**t vs. d**n), and timing of swearing. Finally, future studies should investigate optimal use of clinician swearing, identifying conditions under which swearing may enhance outcomes, and, perhaps more importantly, the contexts in which it may undermine them. Such research would help establish research-supported guidelines for the sensitive and strategic use of swearing in healthcare communication.

### 4.6 Practical recommendations

Although exploratory and underpowered, these findings suggest several recommendations for clinicians considering the use of swearing in physical rehabilitation settings.

1. *When in doubt, don't swear.* It may be impossible to swear indiscriminately with every patient in every situation. Swearing was not universally accepted, and some patients indicated it is never appropriate. Therefore, swearing should not be applied broadly with all patients; restraint is the safest approach.
2. *Use sparingly and with purpose.* Patients reported that swearing was most appropriate when it motivated, encouraged, or built rapport. It should not be a habitual or filler behavior.
3. *Align with patient preferences.* Patients differ widely in tolerance of swearing. Clinicians should gauge comfort and avoid swearing with patients who appear uncomfortable, as well as with younger or older patients, who were specifically identified as groups where swearing is less appropriate.
4. *Never swear in anger or at the patient.* Patients identified swearing in frustration or during serious conversations as inappropriate and potentially harmful.
5. *Prioritize authenticity.* Swearing appears to be most effective when it feels natural, tied to genuine emotion, humor, or solidarity. Forced swearing risks reducing sincerity.

## 5 Conclusion

This exploratory study provides preliminary evidence regarding how patients perceive clinicians' swearing in physical rehabilitation settings. While swearing is often considered inappropriate in professional contexts, this study suggests that swearing may enhance aspects of the rehabilitation process, including patient motivation, self-confidence, adherence, and overall experience of care. Over half of the patients surveyed reported that their clinician used swear words during treatment. Most patients who experienced clinician swearing reported neutral to positive outcomes.

While higher frequency of clinician swearing did not significantly influence motivation, self-confidence, adherence, and patient perceptions of professionalism, offensiveness, or overall benefit to the rehabilitation experience, higher clinician swearing frequencies were associated with greater perceptions of patient-centeredness.

The qualitative findings provided nuanced insight into patients' contextual judgments about clinician swearing. Patients generally viewed swearing as appropriate when it enhanced motivation, humor, or rapport, and inappropriate when it was used in anger, or disregarded patient comfort and demographics. These results suggest that swearing in healthcare may be a potentially effective tool, but one that carries risks if used indiscriminately. Importantly, these findings are preliminary, underpowered, and context-dependent, reinforcing the need for further research before swearing can be recommended as a clinical strategy.

## Data Availability

The datasets presented in this study can be found in online repositories. The names of the repository/repositories and accession number(s) can be found below: https://osf.io/4bu9h/.
